# Supplementary material for: Allosteric regulation and crystallographic fragment screening of SARS-CoV-2 NSP15 endoribonuclease
Source: Nucleic Acids Res. 2023 Apr 28;51(10):5255–70. doi: 10.1093/nar/gkad314 (PMC10250223; doi:10.1093/nar/gkad314)
Supplement: gkad314_Supplemental_Files [file gkad314_supplemental_files.zip › Supplementary datasets.zip!/231/Refine_8/01-BUSTER/Cycle-3/shell.01/R_Rfree.html]

# Rxpct factors in resolution bins at cycle 0


|  |  |  |  |  |  |  |  |  |
| --- | --- | --- | --- | --- | --- | --- | --- | --- |
|  | **<d> (Å)**  Rxpct\_Work | Rxpct\_Free | Rxpct\_All | |  |  |  |  |
|  | 13.1576 | 0.3230 | 0.3739 | 0.3252 |  |
|  | 7.5965 | 0.2667 | 0.2887 | 0.2680 |  |
|  | 5.8843 | 0.2852 | 0.2884 | 0.2853 |  |
|  | 4.9731 | 0.2150 | 0.2485 | 0.2168 |  |
|  | 4.3859 | 0.2024 | 0.1946 | 0.2020 |  |
|  | 3.9672 | 0.2109 | 0.2267 | 0.2116 |  |
|  | 3.6493 | 0.2293 | 0.2688 | 0.2312 |  |
|  | 3.3973 | 0.2329 | 0.2636 | 0.2346 |  |
|  | 3.1912 | 0.2310 | 0.2281 | 0.2309 |  |
|  | 3.0186 | 0.2211 | 0.2317 | 0.2215 |  |
|  | 2.8712 | 0.2195 | 0.2272 | 0.2199 |  |
|  | 2.7435 | 0.2079 | 0.2309 | 0.2090 |  |
|  | 2.6315 | 0.2027 | 0.2330 | 0.2045 |  |
|  | 2.5322 | 0.1928 | 0.1942 | 0.1929 |  |
|  | 2.4433 | 0.1864 | 0.1925 | 0.1867 |  |
|  | 2.3632 | 0.1875 | 0.1879 | 0.1876 |  |
|  | 2.2904 | 0.1844 | 0.1797 | 0.1842 |  |
|  | 2.2240 | 0.1975 | 0.2130 | 0.1982 |  |
|  | 2.1631 | 0.1990 | 0.2214 | 0.2000 |  |
|  | 2.1069 | 0.2431 | 0.2275 | 0.2422 |  |
|  | **Overall** | 0.2265 | 0.2420 | 0.2273 |  |

# Rxpct factors in resolution bins at cycle 1


|  |  |  |  |  |  |  |  |  |
| --- | --- | --- | --- | --- | --- | --- | --- | --- |
|  | **<d> (Å)**  Rxpct\_Work | Rxpct\_Free | Rxpct\_All | |  |  |  |  |
|  | 13.1576 | 0.3278 | 0.3794 | 0.3300 |  |
|  | 7.5965 | 0.2722 | 0.2978 | 0.2738 |  |
|  | 5.8843 | 0.2872 | 0.2937 | 0.2875 |  |
|  | 4.9731 | 0.2143 | 0.2515 | 0.2164 |  |
|  | 4.3859 | 0.2004 | 0.1939 | 0.2000 |  |
|  | 3.9672 | 0.2083 | 0.2255 | 0.2091 |  |
|  | 3.6493 | 0.2278 | 0.2692 | 0.2299 |  |
|  | 3.3973 | 0.2310 | 0.2629 | 0.2327 |  |
|  | 3.1912 | 0.2286 | 0.2255 | 0.2284 |  |
|  | 3.0186 | 0.2195 | 0.2292 | 0.2199 |  |
|  | 2.8712 | 0.2171 | 0.2251 | 0.2175 |  |
|  | 2.7435 | 0.2070 | 0.2316 | 0.2082 |  |
|  | 2.6315 | 0.2030 | 0.2322 | 0.2047 |  |
|  | 2.5322 | 0.1936 | 0.1966 | 0.1938 |  |
|  | 2.4433 | 0.1872 | 0.1959 | 0.1876 |  |
|  | 2.3632 | 0.1888 | 0.1914 | 0.1889 |  |
|  | 2.2904 | 0.1852 | 0.1844 | 0.1851 |  |
|  | 2.2240 | 0.1990 | 0.2101 | 0.1995 |  |
|  | 2.1631 | 0.2002 | 0.2214 | 0.2012 |  |
|  | 2.1069 | 0.2439 | 0.2310 | 0.2432 |  |
|  | **Overall** | 0.2262 | 0.2432 | 0.2271 |  |

# Rxpct factors in resolution bins at cycle 2


|  |  |  |  |  |  |  |  |  |
| --- | --- | --- | --- | --- | --- | --- | --- | --- |
|  | **<d> (Å)**  Rxpct\_Work | Rxpct\_Free | Rxpct\_All | |  |  |  |  |
|  | 13.1576 | 0.3248 | 0.3739 | 0.3269 |  |
|  | 7.5965 | 0.2675 | 0.2939 | 0.2691 |  |
|  | 5.8843 | 0.2825 | 0.2924 | 0.2829 |  |
|  | 4.9731 | 0.2122 | 0.2477 | 0.2143 |  |
|  | 4.3859 | 0.1999 | 0.1936 | 0.1996 |  |
|  | 3.9672 | 0.2087 | 0.2251 | 0.2094 |  |
|  | 3.6493 | 0.2283 | 0.2689 | 0.2303 |  |
|  | 3.3973 | 0.2314 | 0.2622 | 0.2331 |  |
|  | 3.1912 | 0.2281 | 0.2251 | 0.2279 |  |
|  | 3.0186 | 0.2189 | 0.2295 | 0.2193 |  |
|  | 2.8712 | 0.2166 | 0.2217 | 0.2168 |  |
|  | 2.7435 | 0.2055 | 0.2298 | 0.2067 |  |
|  | 2.6315 | 0.2018 | 0.2275 | 0.2034 |  |
|  | 2.5322 | 0.1909 | 0.1945 | 0.1911 |  |
|  | 2.4433 | 0.1851 | 0.1963 | 0.1856 |  |
|  | 2.3632 | 0.1874 | 0.1896 | 0.1875 |  |
|  | 2.2904 | 0.1834 | 0.1808 | 0.1832 |  |
|  | 2.2240 | 0.1992 | 0.2097 | 0.1997 |  |
|  | 2.1631 | 0.2000 | 0.2220 | 0.2010 |  |
|  | 2.1069 | 0.2445 | 0.2326 | 0.2438 |  |
|  | **Overall** | 0.2250 | 0.2415 | 0.2258 |  |

# Rxpct factors in resolution bins at cycle 3


|  |  |  |  |  |  |  |  |  |
| --- | --- | --- | --- | --- | --- | --- | --- | --- |
|  | **<d> (Å)**  Rxpct\_Work | Rxpct\_Free | Rxpct\_All | |  |  |  |  |
|  | 13.1576 | 0.3255 | 0.3775 | 0.3277 |  |
|  | 7.5965 | 0.2692 | 0.3010 | 0.2712 |  |
|  | 5.8843 | 0.2786 | 0.2867 | 0.2789 |  |
|  | 4.9731 | 0.2109 | 0.2500 | 0.2131 |  |
|  | 4.3859 | 0.1979 | 0.1955 | 0.1977 |  |
|  | 3.9672 | 0.2069 | 0.2270 | 0.2079 |  |
|  | 3.6493 | 0.2270 | 0.2682 | 0.2290 |  |
|  | 3.3973 | 0.2295 | 0.2620 | 0.2313 |  |
|  | 3.1912 | 0.2264 | 0.2271 | 0.2264 |  |
|  | 3.0186 | 0.2170 | 0.2261 | 0.2174 |  |
|  | 2.8712 | 0.2153 | 0.2162 | 0.2153 |  |
|  | 2.7435 | 0.2038 | 0.2275 | 0.2050 |  |
|  | 2.6315 | 0.2005 | 0.2255 | 0.2020 |  |
|  | 2.5322 | 0.1894 | 0.1892 | 0.1894 |  |
|  | 2.4433 | 0.1826 | 0.1916 | 0.1830 |  |
|  | 2.3632 | 0.1845 | 0.1887 | 0.1847 |  |
|  | 2.2904 | 0.1813 | 0.1758 | 0.1811 |  |
|  | 2.2240 | 0.1965 | 0.2092 | 0.1971 |  |
|  | 2.1631 | 0.1966 | 0.2174 | 0.1976 |  |
|  | 2.1069 | 0.2376 | 0.2273 | 0.2370 |  |
|  | **Overall** | 0.2234 | 0.2414 | 0.2243 |  |
